# A Low-Molecular-Weight Compound K7174 Represses Hepcidin: Possible Therapeutic Strategy against Anemia of Chronic Disease

**DOI:** 10.1371/journal.pone.0075568

**Published:** 2013-09-27

**Authors:** Tohru Fujiwara, Takashi Ikeda, Yuki Nagasaka, Yoko Okitsu, Yuna Katsuoka, Noriko Fukuhara, Yasushi Onishi, Kenichi Ishizawa, Ryo Ichinohasama, Naohisa Tomosugi, Hideo Harigae

**Affiliations:** 1 Department of Hematology and Rheumatology, Tohoku University Graduate School, Sendai, Japan; 2 Molecular Hematology/Oncology, Tohoku University Graduate School, Sendai, Japan; 3 Clinical Research, Innovation and Education Center, Tohoku University Hospital, Sendai, Japan; 4 Hematopathology, Tohoku University Graduate School, Sendai, Japan; 5 Aging Research Unit, Division of Advanced Medicine, Medical Research Institute, Kanazawa Medical College, Kanazawa, Japan; Queen's University Belfast, United Kingdom

## Abstract

Hepcidin is the principal iron regulatory hormone, controlling the systemic absorption and remobilization of iron from intracellular stores. The expression of the hepcidin gene, *HAMP*, is increased in patients with anemia of chronic disease. Previously, the synthetic compound K7174 was identified through chemical screening as a novel inhibitor of the adhesion of monocytes to cytokine-stimulated endothelial cells. K7174 also ameliorated anemia induced by inflammatory cytokines in mice, which suggests a possible involvement of hepcidin regulation. The present study was performed to assess the impact of K7174 on hepcidin expression in a human hematoma cell line and in mice *in vivo*. We first demonstrated that K7174 treatment in HepG2 cells significantly decreased *HAMP* expression. Then, we conducted microarray analysis to determine the molecular mechanism by which K7174 inhibits *HAMP* expression. Transcriptional profiling confirmed the downregulation of *HAMP*. Surprisingly, we found that K7174 strongly induced GDF15, known as a negative regulator of *HAMP* expression. Western blotting analysis as well as ELISA confirmed the induction of GDF15 by K7174 treatment. Furthermore, K7174-mediated *HAMP* suppression was rescued by the silencing of GDF15 expression. Interestingly, we found that K7174 also upregulates CEBPB. Promoter analysis and chromatin immunoprecipitation analysis revealed that CEBPB could contribute to K7174-mediated transcriptional activation of GDF15. Subsequently, we also examined whether K7174 inhibits hepcidin expression in mice. Quantitative RT-PCR analysis with liver samples from K7174-treated mice demonstrated significant upregulation of *Gdf15* and downregulation of *Hamp* expression, as compared to control mice. Furthermore, serum hepcidin concentration was also significantly decreased in K7174-treated mice. In conclusion, K7174 inhibits hepcidin expression partly by inducing GDF15. K-7174 may be a potential therapeutic option to treat anemia of chronic disease.

## Introduction

Hepcidin is a circulating peptide of 25 amino acids produced by the liver, and is a central hormonal regulator of systemic iron balance [1,2]. Hepcidin binds to ferroportin, a cellular iron exporter that is highly expressed at the enterocyte basolateral membrane and on macrophages, and limits the entry of iron into the plasma by triggering ferroportin internalization and degradation [3]. The expression of *HAMP* encoding hepcidin is regulated in response to iron loading, inflammation, and erythropoietic activity [1,2], and these stimuli modify the quantity and distribution of iron in the body.

Anemia of chronic disease (ACD), also known as anemia of inflammation, is the most prevalent type of anemia in hospitalized patients worldwide [4]. The pathogenesis of ACD is characterized by iron-restricted erythropoiesis, whereas iron is retained in the macrophages and there may be an increase in total body iron [4,5]. It has now become clear that inflammatory cytokines released during acute infection or chronic disease can alter systemic iron metabolism by inducing excess synthesis of hepcidin [6-8]. Treatment of anemia, when necessary, has included administration of iron, packed red cell transfusion, or erythropoiesis-stimulating agents. However, concerns over adverse effects of these therapies, including iron overload, increased risk of infection, recurrence of cancer, and cardiovascular complications, have driven the need for alternative treatments [5,9,10]. Due to the central role of hepcidin as described above, inhibition of its biological activity may be a promising new approach for the treatment of anemia associated with inflammation.

A previous study indicated that K7174, a synthetic low molecular weight compound that acts as a GATA-specific inhibitor, has the potential to attenuate expression of vascular cell adhesion molecule 1 (VCAM-1) in cytokine-stimulated endothelial cells, which was mediated by the inhibition of GATA factor binding at the VCAM-1 gene promoter region [11]. Another study demonstrated that K7174 inhibited GATA-2-mediated negative regulation for erythropoietin gene, which might contribute to the amelioration of anemia induced by inflammatory cytokines in mice [12]. However, although the latter report suggested the possible presence of ACD [12], the involvement of hepcidin regulation was not described. Thus, the present study was performed to investigate the effects of K7174 on hepcidin expression *in vitro* and *in vivo*.

## Materials and Methods

### Cell culture

Cells were grown in a humidified incubator at 37°C with 5% CO_2_. Human HepG2 hematoma cells and the PLAT-GP Packaging Cell Line (Cell Biolabs) [13] were maintained in DMEM containing 10% fetal bovine serum (Biowest) and 1% penicillin-streptomycin (Sigma). Human K562 erythroleukemia cells [13] were maintained in RPMI-1640 medium containing 10% fetal bovine serum (Biowest) and 1% penicillin-streptomycin (Sigma). HepG2 and K562 cells were treated with 10 µM or 20 µM K7174 (Kowa, Tokyo, Japan) for 24 h [11,12]. For K7174 or BMP4 treatment, both HepG2 and K562 were seeded at 1 x 10^6^ cells/100 mm dish on the day before treatment. HepG2 cell line was obtained from Cell Resource Center for Biomedical Research, Tohoku University (www2.idac.tohoku.ac.jp/dep/ccr/). Recombinant human BMP4 was obtained from R&D systems (MN, USA).

### Real-time quantitative RT-PCR

Total RNA was purified with TRIzol (Invitrogen), and 1 µg of purified total RNA was used to synthesize cDNA with ReverTra Ace qPCR RT Master Mix with gDNA Remover (Toyobo), which can exclude genomic DNA from total RNA samples before cDNA synthesis. Reaction mixtures (20 µL) for real-time quantitative RT-PCR consisted of 2 µL of cDNA, 10 µL of Quantitect SYBR, Green (Qiagen), and appropriate primers. Primer sequences are listed on Table S1. Product accumulation was monitored by measuring SYBR Green fluorescence and normalized relative to GAPDH mRNA [13,14].

### Microarray analysis

For expression profiling, Human Oligo chip 25 k (Toray) was used essentially as described previously [15]. Briefly, aliquots of 1 µg of total RNA were amplified with an Ambion Amino Allyl aRNA kit (Ambion), labeled with Amersham Cy5 Mono-Reactive Dye (GE Healthcare), and hybridized to the Human Oligo chip 25 k array. Data were collected and normalized using a 3D-Gene Scanner 3000 system (Toray). For global normalization, background value was subtracted, and subsequently adjusted to the average signal value of 25. Each probe on the microarray was linked with specific Gene Ontology (GO) terms based on Oligo MicroArray DataBase system (OMAD) (Operon). Pathway analysis was conducted based on GenMAPP Ver 2.1 (MAPP Finder) (http://www.genmapp.org/). Gene array datasets have been deposited in the GEO database, www.ncbi.nlm.nih.gov/geo (accession no. GSE48618).

### Viral vectors and cell transduction

Retroviral overexpression of GDF15 was conducted using pBABE-puro vector (Addgene Plasmid 1764) [13,16]. The retroviral vector encoding human GDF15 and the *env* (envelope glycoprotein) gene from the vesicular stomatitis virus (VSV-G) were cotransfected into the PLAT-GP Packaging Cell Line (Cell Biolabs) with FuGene HD (Promega). Seventy-two hours after transfection, the viral supernatant was used for infection. After infection into HepG2 cells for 6 h, the cells were incubated with culture medium containing 1 µg/mL Puromycin (Sigma) for selection of transduced cells.

### Silencing of GDF15 gene expression by small interfering RNA (siRNA)

For siRNA-mediated transient knockdown in K562 cells, siGENOME SMART pool (Thermo Scientific Dharmacon, Lafayette, CO) was used. The antisense sequences of the siRNA for human GDF15 were GGGAAGAUUCGAACACCGA, GAGAGUUGCGGAAACGCUA, CGGCAAACAUGCACGCGCA, and GGGUGUCGCUCCAGACCUA. As a negative control, siGENOME Non-targeting siRNA pool 1 (Thermo Scientific Dharmacon) was used. siRNA was transfected into HepG2 cells using Lipofectamine^TM^ RNAiMAX (Invitrogen). Twelve hour after K7174 or DMSO treatment in HepG2 cells in 100 mm dish, 240 pmol of siRNA was transfected once and the cells were harvested at 24 h.

### Promoter assay

The DNA fragment of the human *GDF15* gene promoter region, which contains the *GDF15* promoter (spanning from -1064 to +44) and artificial restriction enzyme sites on both ends (*Xho*I and *Bgl*II, respectively), was artificially synthesized (Medical & Biological Laboratories). The genomic DNA sequence was obtained from the UCSC genome browser (http://genome.ucsc.edu/). The synthesized DNA fragment was digested with *Xho*I and *Bgl*II, and subsequently inserted into the corresponding sites of the pGL4.10 [*luc2*] vector (Promega). To generate deletion constructs, the following primers were used: Forward; 5’-TTTTTTCTCGAGAGGAAACAGGCATGGCAGAGA-3’ (-464), 5’-CCTCCCCCTCGAGACACCCCCAGACCCCGCCCA-3’ (-137), 5’-AGGGCGGGCTCGAGCAGGCGGAGACGGACAAAGTCC-3’ (-64), Reverse; 5’-CGTCCTGAGATCTTGCCCGGGCATGGCTGTGCA-3’(+44) (*Xho*I and *Bgl*II sites are underlined). The amplified PCR fragments were digested with *Xho*I and *Bgl*II, and cloned into the pGL4.10 [*luc2*] vector. For generating mutation constructs, a QuikChange Site-Directed Mutagenesis Kit (Agilent, Santa Clara, CA) was used.

To assay *GDF15* transcriptional activity, aliquots of HepG2 cells were transfected with 1 µg of *GDF15* promoter construct and 100 ng of the pGL4.74 [*hRluc*/TK] vector (Promega), with FuGene HD (Promega). On the day before plasmid transfection (day -1), the HepG2 cells were seeded using a 6 well plate (Becton Dickinson and company, NJ, USA) at 5 x 10^5^ cells/well. Twenty-four hour after plasmids transfection, the cells were harvested, and both firefly and *Renilla* luciferase activities in the cell extracts were determined using the Dual Luciferase Reporter Assay System (Promega). K7174 treatment was performed 4 h after plasmid transfection at a final concentration of 20 µM.

### Western blotting analysis

Whole-cell extracts were prepared by boiling cells for 10 min in SDS sample buffer [25 mM Tris (pH 6.8), 2% beta-mercaptoethanol, 3% SDS, 0.1% bromophenol blue, 5% glycerol] at 1×10^7^ cells/mL. Extracts of 1-2×10^5^ cells were resolved by SDS-PAGE and transferred onto Hybond-P polyvinylidene difluoride (PVDF) membranes (GE Healthcare). The proteins were measured semiquantitatively with ECL-Plus (GE Healthcare) and CL-X Posure™ Film (Thermo Scientific).

### Quantitative ChIP analysis

Real-time PCR-based quantitative chromatin immunoprecipitation (ChIP) analysis was conducted essentially as described [14]. Cells were crosslinked with 1% formaldehyde for 10 min at room temperature. The nuclear lysate was sonicated to reduce DNA length using a Sonifier (Branson). The protein-DNA complexes were immunoprecipitated by specific antibody and Protein A or G Sepharose (Sigma). Immunoprecipitated DNA fragments were quantified by real-time PCR to amplify regions of 75-150 bp overlapping with the appropriate motif. Product was measured by SYBR Green fluorescence in 20 µL reactions, and the amount of product was determined relative to a standard curve generated by titration of input chromatin. Analysis of postamplification dissociation curves showed that primer pairs generated single products. Primer sequences are listed on Table S1.

### Antibodies

Antibodies to GATA-4 (C-20) and C/EBPbeta (C-19) were obtained from Santa Cruz Biotechnology. Alpha-Tubulin (CP06) was obtained from Calbiochem. Control IgG (rabbit and goat) and GDF15 (ab14586) were obtained from Abcam. Smad1 and Phospho-Smad1(Ser463/465)/Smad5(Ser463/465)/Smad8(Ser426/428) were obtained from Cell Signaling Technology (MA, USA).

### ELISA (Enzyme-linked immunosorbent assay)

Enzyme-linked immunosorbent assay (ELISA) was performed to measure human GDF15 concentration with a Quantikine ELISA kit for human GDF15 (R&D Systems).

### In vivo mouse analysis

The protocol for *in vivo* analysis in mice was based on the previous study [12], with minor modifications. Briefly, ICR mice were injected intraperitoneally once per day with PBS (control) or 30 mg/kg K7174, respectively, on days 0, 1, 2, 3, 5, 6, 7 and 8, and samples were obtained on day 9 under anesthetic condition with diethyl ether (Wako, JAPAN). The study was conducted at Institute for Animal Experimentation, Tohoku University Graduate School of Medicine, and was approved by approved by the ethical committee of Tohoku University.

### Quantitative analysis of hepcidin-25

Sera from all mice were frozen and stored at -80°C until analysis. Serum hepcidin-25 levels were determined using a liquid chromatography–tandem mass spectrometry (LC-MS/MS)-based assay system as described previously [17].

### Statistics

Statistical significance was assessed by two-tailed Student’s *t* test and one-way analysis of variance (ANOVA) analysis. For pathway analysis, the terms showing Z score > 2 and *P* < 0.05 were selected and ranked. In all analyses, *P* < 0.05 was taken to indicate statistical significance.

## Results and Discussion

### K7174 treatment suppresses HAMP expression in HepG2 cells

**Figure 1:**
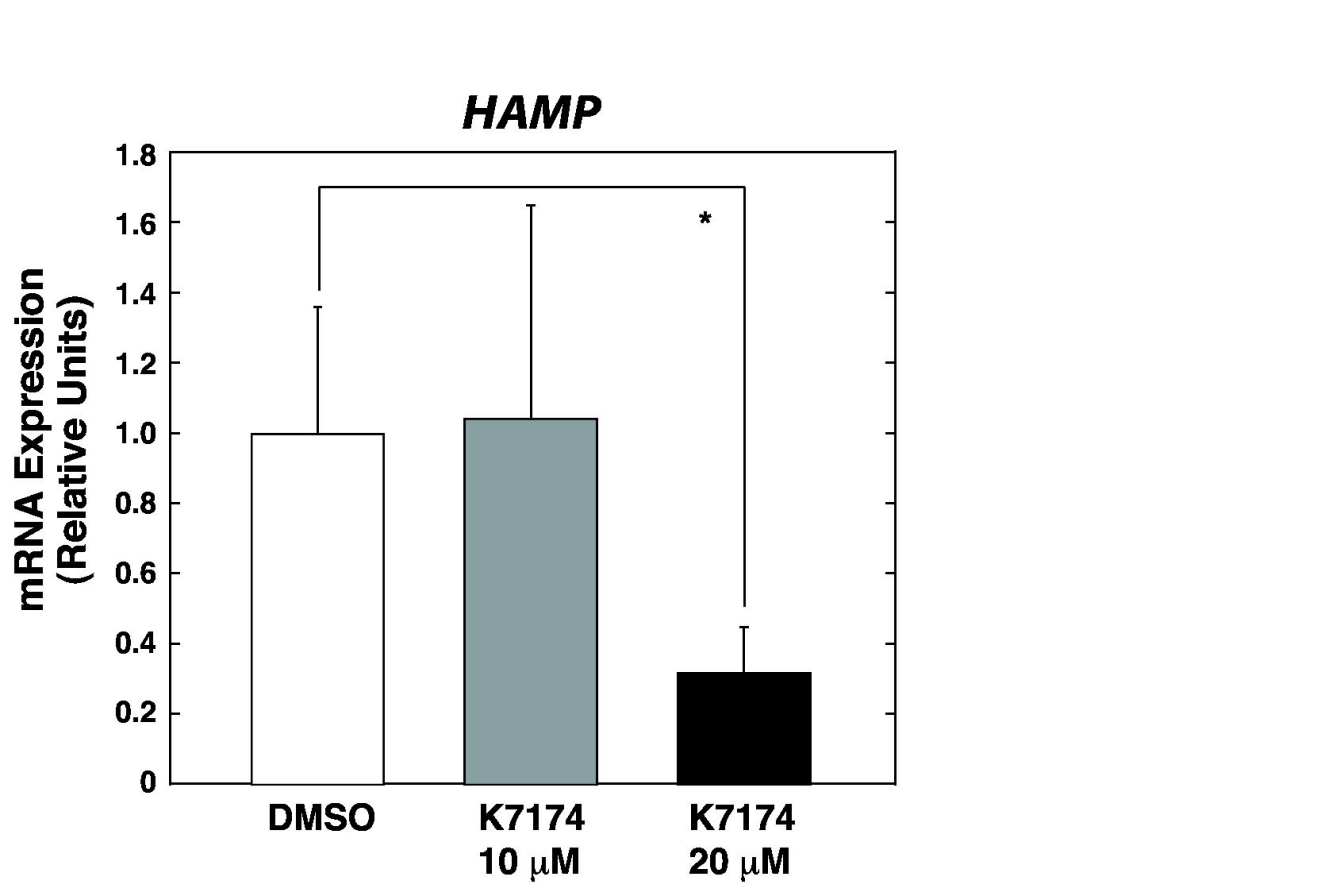
K7174 suppresses HAMP expression in HepG2 cells. Quantitative RT-PCR analysis of *HAMP* expression in control (DMSO) or K7174-treated HepG2 cells. The expression level of each target gene relative to that of *GAPDH* was calculated (*n* = 5, mean ± SE, * *P* < 0.05). The expression level of DMSO-treated control cells was set to 1.

First, to examine whether K7174 affects *HAMP* expression, HepG2 cells were treated with 10 or 20 µM K7174 or DMSO as a control. The K7174 concentration was determined based on the method described previously [12]. As shown in Figure 1, treatment with 20 µM K7174 for 24 h led to significant downregulation of *HAMP* expression, whereas ANOVA analysis among 3 groups failed to yield the statistical significance (p = 0.83). As K7174 was originally identified as a GATA-specific inhibitor [11,12], and *HAMP* expression has been reported to be regulated by one of the GATA factors, GATA-4, through the GATA-binding element located in its promoter region in HepG2 cells [18], we initially postulated that K7174 may reduce *HAMP* expression by affecting GATA-4 chromatin occupancy at the *HAMP* promoter. However, K7174 treatment did not alter GATA-4 chromatin occupancy (Figure S1). We also examined GATA-2 chromatin occupancy under the same conditions using anti-GATA-2 antibody [15], but found no detectable GATA-2 signal compared to control IgG (data not shown), presumably reflecting low expression level of GATA-2. These data suggest that other regulatory mechanisms may be involved in the K7174-mediated repression of *HAMP* transcription.

### Transcriptional profiling identified GDF15 as one of the causative genes contributing to K7174-mediated HAMP repression

**Figure 2:**
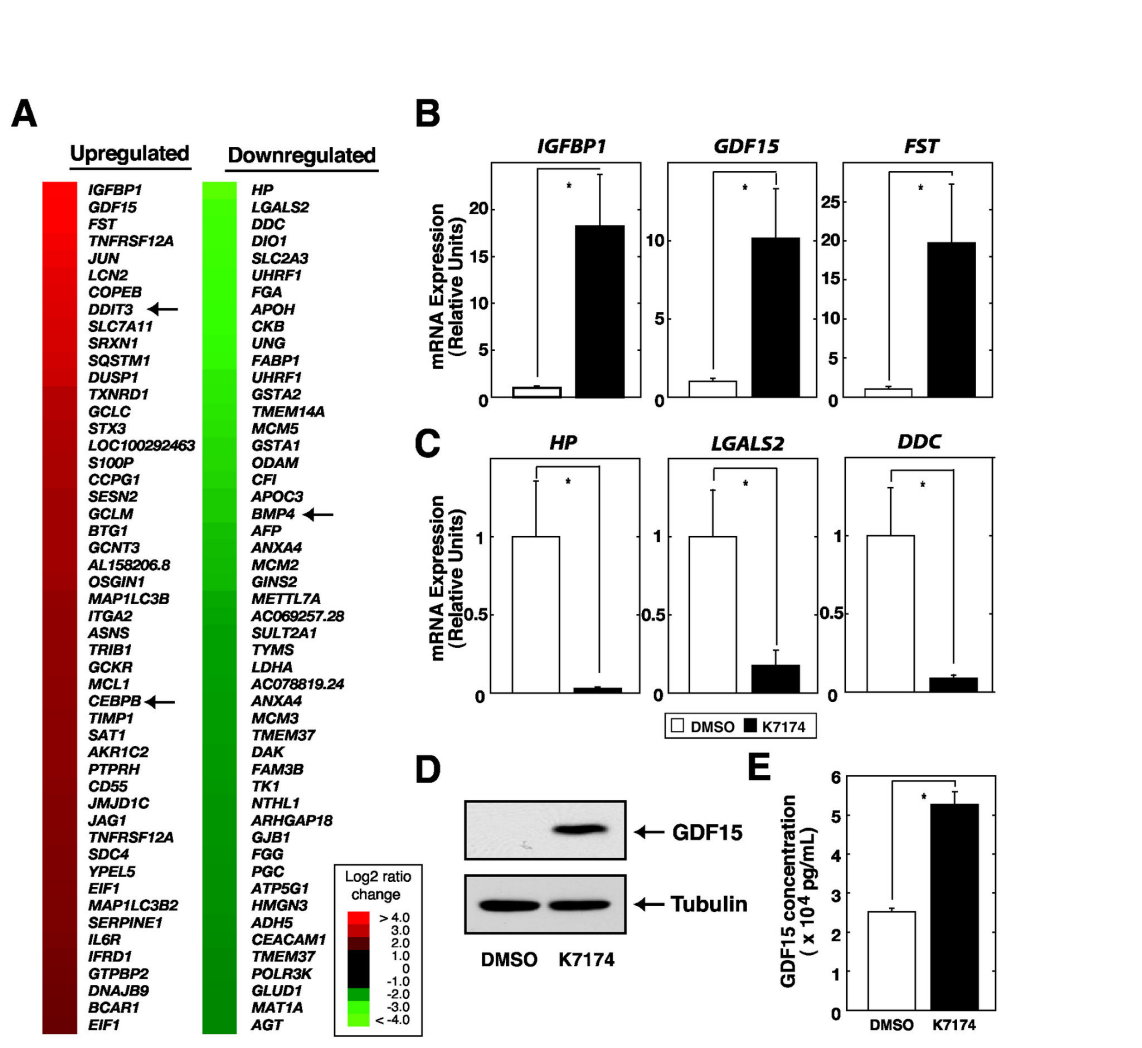
Expression profiling identified GDF15 among K7174-regulated genes. (A) Expression profiling results based on HepG2 cells treated with K7174 (20 µM, 24 h) or 0.1% DMSO as a control. The heat map depicts the fold change resulting from K7174 treatment. The top 50 upregulated (left) and downregulated (right) genes after K7174 treatment are shown. Arrows indicate the positions of *DDIT3* (CHOP), *CEBPB* and *BMP4*. (B,C) Quantitative RT-PCR validation of array results for upregulated (B) and downregulated (C) genes. The expression level of each target gene relative to that of *GAPDH* was calculated (*n* = 5, mean ± SE, * *P* < 0.05). The expression levels of DMSO-treated control cells were set to 1. (D) Western blotting analysis of whole-cell extracts from control (DMSO) or K7174-treated HepG2 cells. Anti-GDF15 antibody was used to detect endogenous GDF15 protein. Alpha-Tubulin was used as a loading control. (E) ELISA to measure GDF15 concentration in culture media from control (DMSO) or K7174-treated HepG2 cells (*n* = 5, mean ± SE, * *P* < 0.05).

To explore the molecular mechanisms by which K7174 suppresses *HAMP* expression, we next compared gene expression profiles of HepG2 cells treated with DMSO or 20 µM K7174 for 24 h. The analysis revealed 1311 and 1284 genes that were upregulated and downregulated (> 1.5 fold), respectively, in K7174-treated cells (Figure 2A, Table S2). We confirmed the inclusion of the *HAMP* gene among K7174-downregulated genes (1.83-fold downregulation) (Table S2), which was similar to the results of quantitative RT-PCR (Figure 1). On the other hand, however, our profiling analysis did not include erythropoietin gene among K7174 regulated gene ensemble, which was reported to be regulated through the GATA factor inhibition [12] (Table S2). Quantitative RT-PCR-based validation analysis for the top three regulated genes demonstrated obvious upregulation of *IGFBP1*, *GDF15*, and *FST* (Figure 2B) as well as downregulation of *HP*, *LGALS2*, and *DDC* (Figure 2C). Interestingly, recent evidence has suggested that GDF15, a member of the TGF-beta cytokines, blocks hepcidin expression, and therefore contributes to iron overload syndrome in thalassemia patients [19]. Therefore, we focused on GDF15 as a candidate molecule that would contribute to the K7174-mediated *HAMP* suppression. Western blotting analysis and ELISA with culture media confirmed the increase in GDF15 protein level in K7174-treated cells (Figure 2D, 2E). Furthermore, pathway analysis revealed significant involvement of TGF-beta signaling (Table 1), suggesting that K7174 has a significant effect on GDF15 signaling in HepG2 cells.

**Table 1:** Pathway Analysis of K7174-regulated genes.

	MAPP Name	N	Z Score
(Upregulated)	Senescence_and_Autophagy	13	4.57
	Phosphatidylinositol_signaling_system	11	3.84
	Folate	8	3.81
	Keap1-Nrf2	3	3.5
	MAPK_signaling_pathway	12	3.3
	Selenium	7	2.93
	Glutathione_metabolism	3	2.89
	Glycine_serine_and_threonine_metabolism	5	2.78
	Adipogenesis	10	2.7
	Cysteine_metabolism	2	2.36
	Keratan_sulfate_biosynthesis	2	2.36
	Ganglioside_biosynthesis	2	2.36
	Insulin_Signaling	11	2.35
	Glutamate_metabolism	4	2.32
	TGF_Beta_Signaling_Pathway	5	2.32
	Type_II_interferon_signaling_(IFNG)	3	2.25
	Regulation_of_Actin_Cytoskeleton	9	2.21
	Nuclear_receptors_in_lipid_metabolism_and_toxicity	3	2.09
	Nitrogen_metabolism	2	2.08
(Downregulated)	DNA_Replication	16	7.45
	G1_to_S_cell_cycle_control	15	5.04
	Electron_Transport_Chain	20	4.61
	Oxidative_phosphorylation	14	4.45
	Mismatch_repair	4	4.09
	Cell_cycle	15	3.96
	Urea_cycle_and_metabolism_of_amino_groups	6	3.8
	beta_Alanine_metabolism	5	3.59
	Folate	9	3.46
	Fatty_acid_metabolism	9	3.35
	Arginine_and_proline_metabolism	9	3.24
	D_Glutamine_and_D_glutamate_metabolism	2	3.22
	Methane_metabolism	3	3.21
	Nucleotide_Metabolism	5	3.14
	Urea_cycle_and_metabolism_of_amino_groups	5	3.14
	Lysine_degradation	8	2.98
	One_carbon_pool_by_folate	4	2.86
	Fatty_acid_biosynthesis_path_2	4	2.86
	Nitrogen_metabolism	3	2.84
	Selenium	8	2.66
	Tryptophan_metabolism	9	2.66
	Statin_Pathway_PharmGKB	5	2.61
	Butanoate_metabolism	6	2.51
	One_Carbon_Metabolism	5	2.46
	Histidine_metabolism	6	2.39
	Bile_acid_biosynthesis	5	2.31
	Ascorbate_and_aldarate_metabolism	3	2.28
	Propanoate_metabolism	5	2.18
	Ubiquinone_biosynthesis	9	2.17
	Glutathione_metabolism	4	2.1
	Glycolysis_Gluconeogenesis	6	2.06
	Glycine_serine_and_threonine_metabolism	5	2.05

Genes showing > 2 fold differences after K7174 treatment in HepG2 cells were analyzed with GenMAPP ver.2.1 (MAPP Finder).

**Figure 3:**
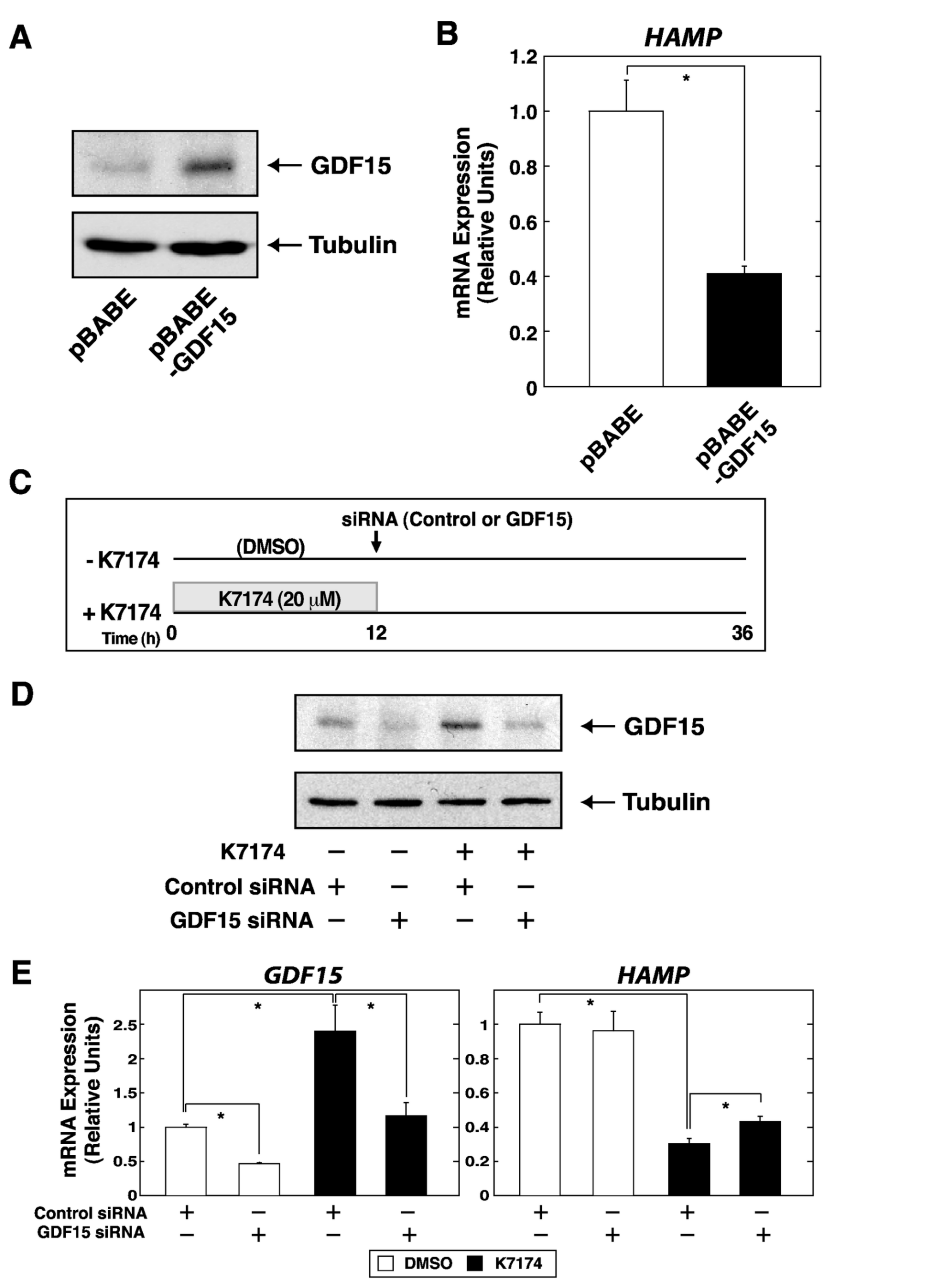
*GDF15 is responsible for K7174-mediated* HAMP *gene downregulation in HepG2 cells.* (A) Retrovirus-mediated GDF15 overexpression in HepG2 cells. Anti-GDF15 antibody was used to detect endogenous GDF15 protein. Alpha-Tubulin was used as a loading control. (B) Quantitative RT-PCR analysis of *HAMP* expression in GDF15 overexpressed HepG2 cells. The expression level of each target gene relative to that of *GAPDH* was calculated (*n* = 3, mean ± SE, * *P* < 0.05). The expression level of DMSO-treated control cells was set to 1. (C) Experimental strategy for siRNA-mediated GDF15 knockdown after K7174 treatment in HepG2 cells. (D) Western blotting analysis of whole-cell extracts from GDF15-silenced HepG2 cells, treated with K7174 or DMSO. Anti-GDF15 antibody was used to detect endogenous GDF15 protein. Alpha-Tubulin was used as a loading control. (E) Quantitative RT-PCR analysis of *GDF15* and *HAMP* expression in GDF15-silenced HepG2 cells, treated with K7174 or DMSO. The expression level of each target gene relative to that of *GAPDH* was calculated (*n* = 3, mean ± SE, * *P* < 0.05). The expression levels of DMSO- and control siRNA-treated control cells were set to 1.

A previous study indicated that GDF15 suppresses *HAMP* expression in hepatocytes using primary hepatocytes as well as the HuH-7 hematoma cell line [19]. We examined whether the result could be confirmed in HepG2 cells used in the present study. Retroviral-mediated overexpression of GDF15 led to the significant downregulation of *HAMP* level in these cells (Figure 3A, 3B). Furthermore, to address if the increase of GDF15 induced by K7174 treatment is truly responsible of the *HAMP* downregulation, we silenced GDF15 expression by siRNA during K7174 treatment (Figure 3C). As shown in Figure 3D and 3E, we confirmed that *GDF15* expression was induced by K7174 treatment, which was inhibited by siRNA against GDF15. Under these conditions, quantitative RT-PCR analysis demonstrated that K7174-mediated *HAMP* suppression was significantly re-activated by siRNA-mediated GDF15 knockdown (Figure 3E). The reason why the rescue of *HAMP* expression after GDF15 knockdown in K7174-treated cells remained partial might be due to the limited GDF15 knockdown efficiency in our experiment, or perhaps co-involvement of other regulatory pathway in K7174-mediated *HAMP* suppression.

### K7174 enhances C/EBPbeta (CEBPB), which directly activates GDF15 transcription

**Figure 4:**
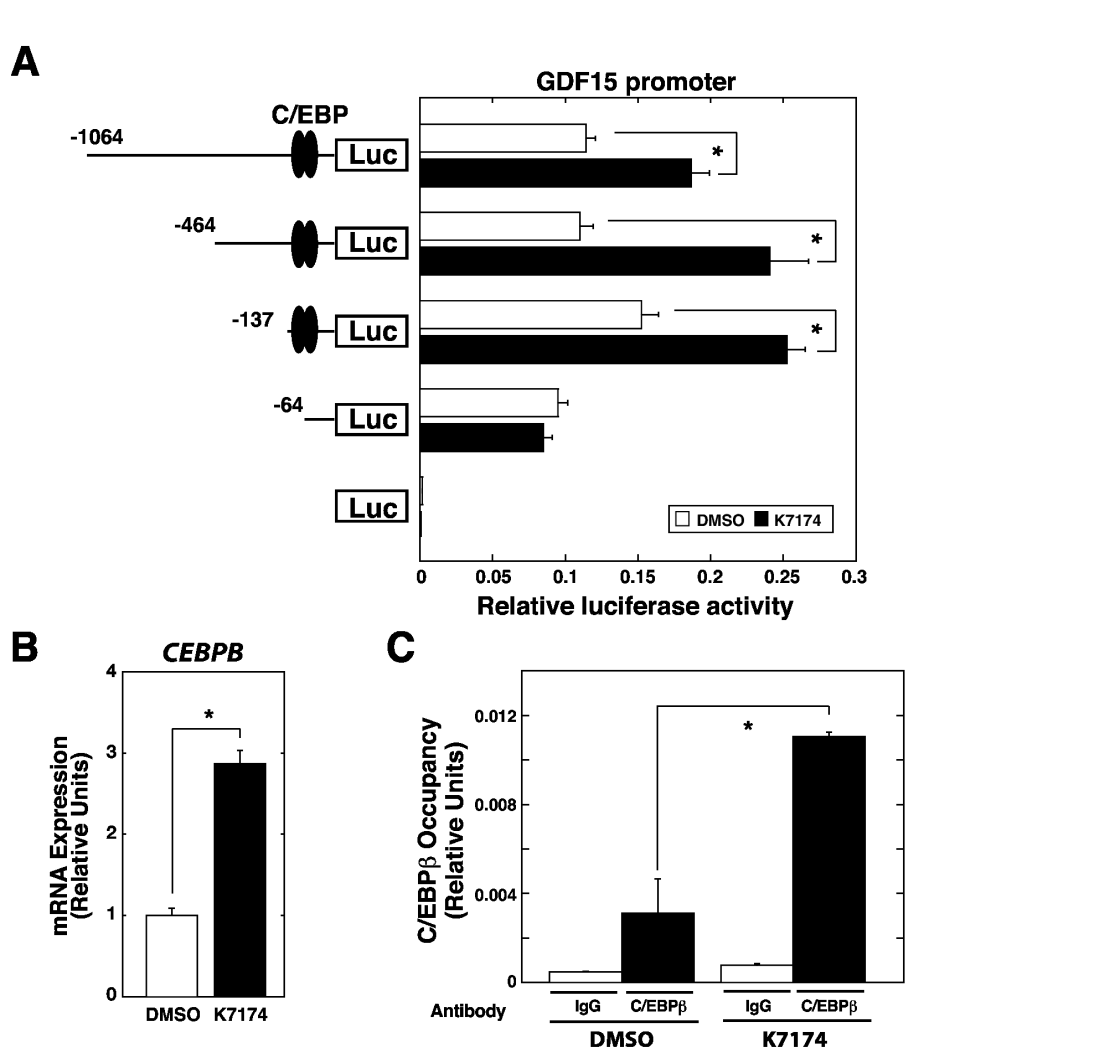
C/EBP binding sites in the GDF15 promoter are important for K7174-mediated transcriptional activation. (A) Transient human *GDF15* gene promoter analysis in control (DMSO) or K7174-treated HepG2 cells. A series of 5’ deletion mutants of the human GDF15 gene promoter were assayed for luciferase activity (*n* = 3, mean ± SE, * *P* < 0.05). (B) Quantitative RT-PCR analysis of *HAMP* expression in control (DMSO) or K7174-treated HepG2 cells. The expression level of each target gene relative to that of *GAPDH* was calculated (*n* = 3, mean ± SE, * *P* < 0.05). The expression level of DMSO-treated control cells was set to 1. (C) Quantitative ChIP analysis in control (DMSO) or K7174-treated HepG2 cells to detect CEBPB chromatin occupancy at GDF15 promoter (*n* = 3, mean ± SE, * *P* < 0.05).

Luciferase promoter analysis was performed to elucidate how *GDF15* transcription is activated by K7174 treatment in HepG2 cells. A previous study indicated that the DNA sequence from –137 to -64 contains putative binding sites for CCAAT/enhancer-binding protein (C/EBP), Sp1, and RARalpha [20,21], and C/EBPbeta (CEBPB) binding to two putative C/EBP sites has a pivotal role in activating capsaicin-induced *GDF15* transcription in colorectal cancer cells [20], implying that these factors may have an important role in the GDF15 promoter activation in HepG2 cells. As shown in Figure 4A, whereas deletion of the sequence from –1064 to -137 did not show significant changes in the promoter activity in DMSO-treated control cells, the promoter activity was significantly induced by K7174 treatment. Further deletion from –137 to -64, containing two putative C/EBP binding sites, still did not markedly reduce the promoter activity, whereas this deletion completely abolished K7174-mediated induction of the promoter activity (Figure 4A). We further generated the CEBPB-binding element-deleted constructs [20], and demonstrated that the deletion of either proximal or distal binding element for CEBPB resulted in the significant decrease in the baseline promoter activity (Figure S2). However, the deletions were not enough to abolish K7174-mediated induction of the luciferase activity (data not shown). Interestingly, we found the upregulation of *CEBPB* among the expression profiling data (Figure 2A), which was also confirmed by quantitative RT-PCR analysis (Figure 4B). In addition, we demonstrated that CEBPB chromatin occupancy at the GDF15 promoter was significantly increased by K7174 treatment (Figure 4C). These data suggest that the upregulation of CEBPB by K7174 contributes to transcriptional activation of GDF15, but it may not be the sole factor responsible for K7174-mediated GDF15 induction.

The mechanisms underlying the induction of *CEBPB* by K7174 are unclear. To our knowledge, there have been no reports of evidence linking GATA inhibition and *CEBPB* upregulation. One possible explanation is that K7174 may induce endoplasmic reticulum (ER) stress [22,23], in addition to its GATA-inhibiting properties [11,12], and the induction of ER stress may trigger CEBPB expression [24]. In support of this observation, we found marked (> 10-fold) upregulation of *DDIT3* based on expression profiling data (Figure 2A). DDIT3 is also known as C/EBP homologous protein (CHOP), which has been reported to be upregulated in the presence of ER stress [22,23]. However, the pathway analysis did not show the significant involvement of ER stress pathway (Table 1). Thus, further analyses are required to determine the role of K7174 in the *CEBPB* regulation.

### BMP-SMAD signaling is not essential in K7174-mediated HAMP repression

**Figure 5:**
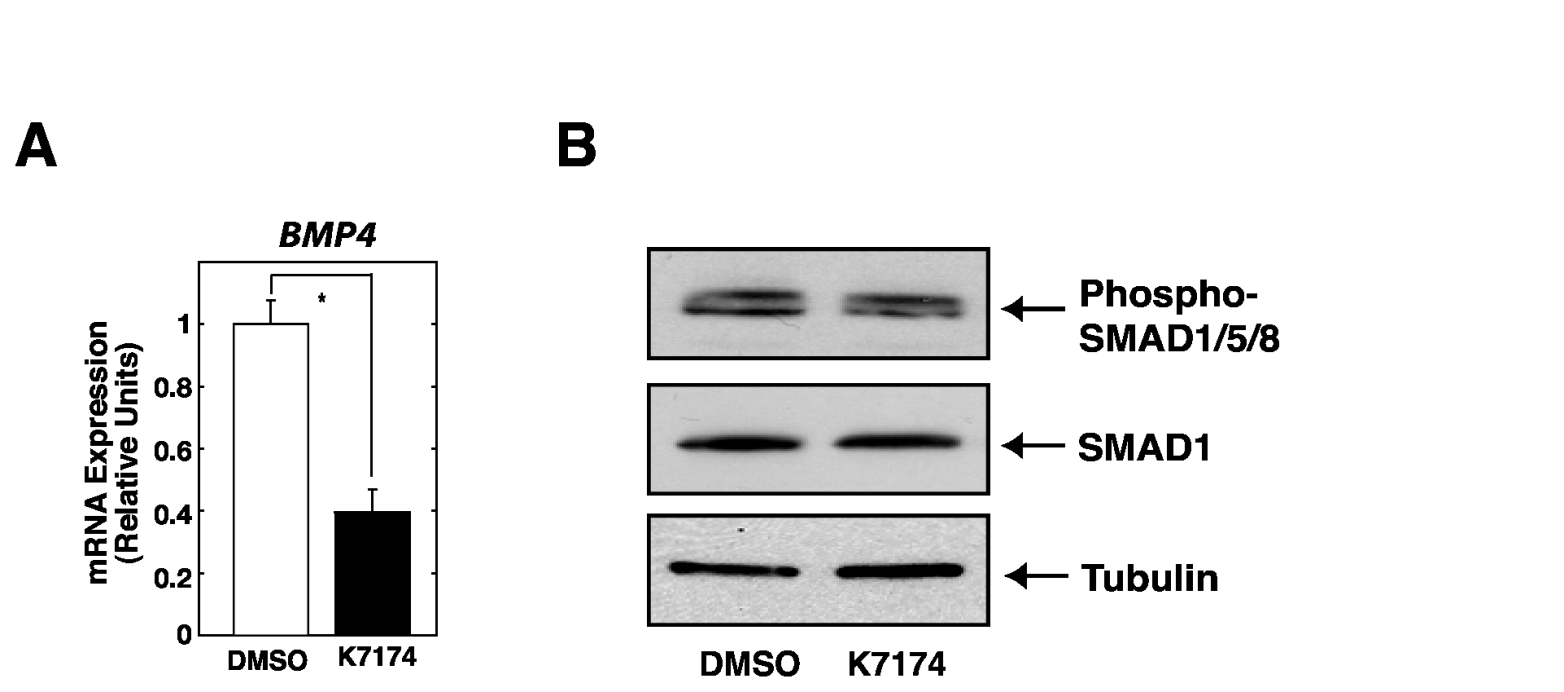
BMP-SMAD signaling was not involved in K7174-mediated suppression of *HAMP*. (A) Quantitative RT-PCR validation of array results for *BMP4* gene. The expression level of each target gene relative to that of *GAPDH* was calculated (*n* = 5, mean ± SE, * *P* < 0.05). The expression level of DMSO-treated control cells was set to 1. (B) Western blotting analysis of whole-cell extracts from K7174- or DMSO-treated HepG2 cells. Anti-phospho-SMAD1/5/8 and SMAD1 antibodies were used. Alpha-Tubulin was used as a loading control.

Our microarray also identified BMP4 among K7174-downregulated gene ensemble (Figures 2A, 5A). As several reports have suggested that BMP4 stimulates *HAMP* expression via activating SMAD signaling [25–27], we assessed the level of SMAD1/5/8 phosphorylation in K7174-treated HepG2 cells. First, we confirmed that the treatment of recombinant BMP4 actually induced SMAD1/5/8 phosphorylation and *HAMP* expression in HepG2 cells (Figure S3). As shown Figure 5B, we did not detect obvious difference in the level of SMAD1/5/8 phosphorylation in K7174-treated HepG2 cells. Although we could not exclude the possibility that we might fail to detect the subtle attenuation of the BMP signal by K7174 treatment, we believe that BMP-SMAD signaling is not essential in K7174-mediated *HAMP* suppression.

### K7174 treatment induces Gdf15 and represses hepcidin expression in mice

**Figure 6:**
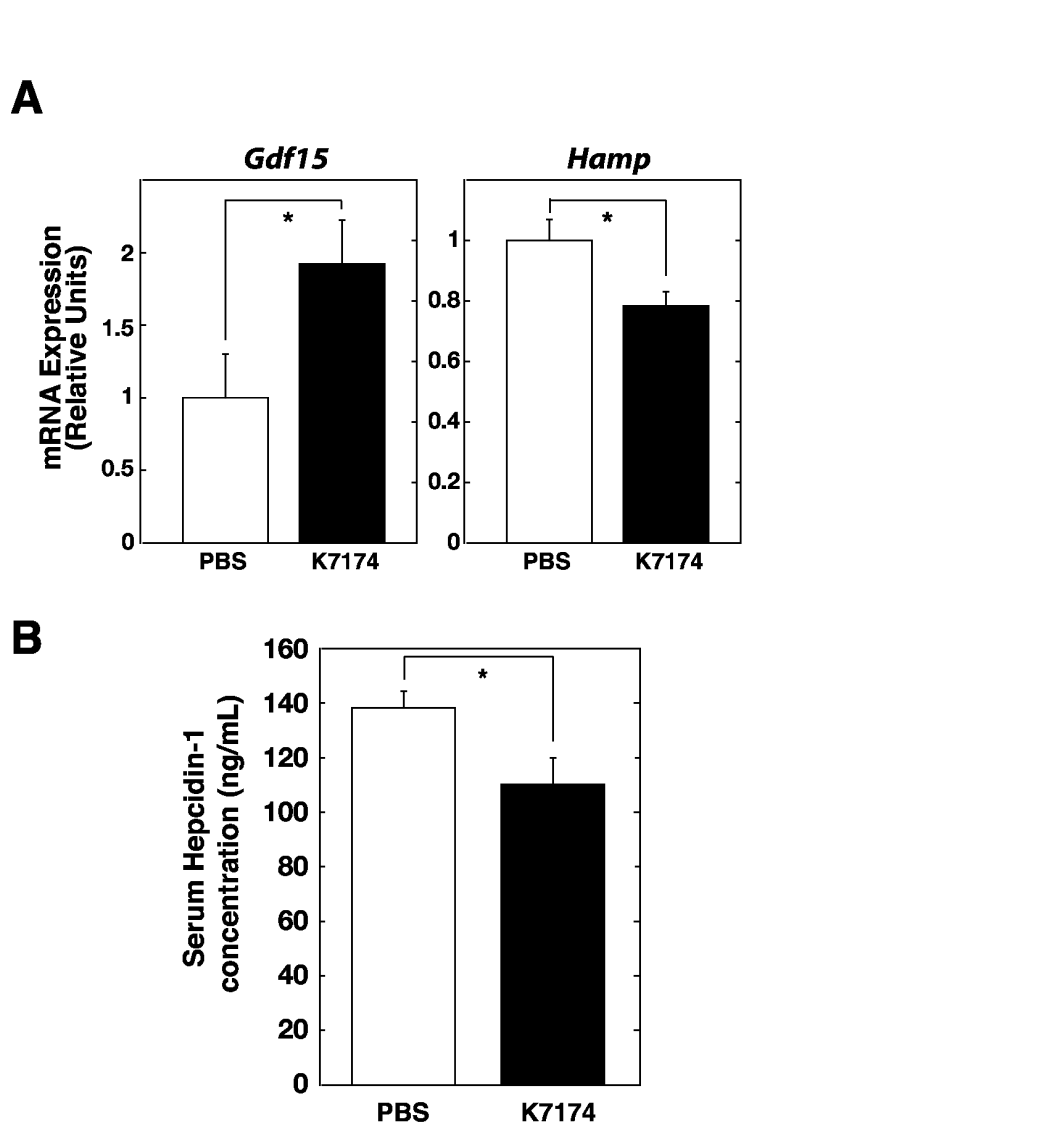
K7174 treatment suppresses hepcidin production *in vivo*. (A) Quantitative RT-PCR analysis of *Gdf15* and *Hamp* expression in liver samples from control (PBS) or K7174-treated mice (*n* = 8, mean ± SE, * *P* < 0.05). The expression levels of PBS-treated liver samples were set to 1. (B) Serum hepcidin (hepcidin1) levels in control (PBS) or K7174-treated mice (*n* = 8, mean ± SE, * *P* < 0.05).

**Figure 7:**
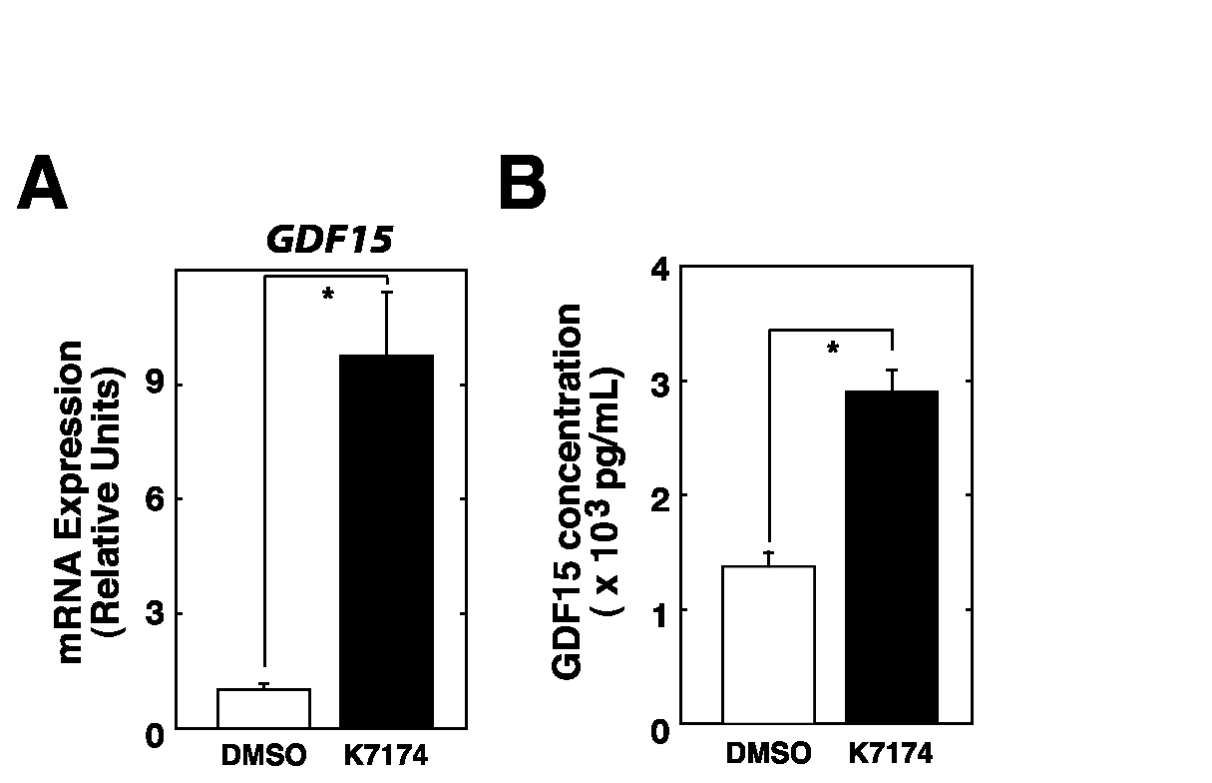
K7174 induced GDF15 in the K562 hematopoietic cell line. (A) Quantitative RT-PCR analysis of GDF15 in control (DMSO) or K7174-treated K562 cells (*n* = 3, mean ± SE, * *P* < 0.05). The expression level of DMSO-treated control cells was set to 1. (E) ELISA to measure GDF15 concentration in culture media from control (DMSO) or K7174-treated K562 cells (*n* = 3, mean ± SE, * *P* < 0.05).

To elucidate the effects of K7174 on GDF15 and hepcidin levels *in vivo*, liver and blood samples from control and K7174-treated mice were obtained. As expected, quantitative RT-PCR analysis of mouse liver samples demonstrated significant upregulation of *Gdf15* and downregulation of *Hamp* (Figure 6A). We also examined serum GDF15 concentration by ELISA, and the results indicated that K7174-treated mice showed higher GDF15 level than controls but the difference was not statistically significant (data not shown). Furthermore, LC-MS/MS analysis demonstrated a significant decrease in serum hepcidin level (Figure 6B), suggesting that increased K7174-mediated induction of GDF15 might suppress hepcidin production in mice. Beyond the regulation of *Gdf15* in hepatocytes, erythroid cells have also been suggested to be one of the main sources of GDF15, especially in conditions associated with inefficient erythropoiesis, such as thalassemia [19]. Therefore, we also examined whether GDF15 could be induced by K7174 in erythroid cells using the K562 erythroid cell line in the present study. As shown in Figure 7, treatment with 20 µM K7174 for 24 h resulted in significant increases in *GDF15* mRNA and protein levels in K562 cells, suggesting that systemic administration of K7174 may act on hepatocytes as well as erythroid cells to stimulate GDF15 production.

In the present study, there were no significant differences in biochemical data, including serum iron, total iron-binding capacity (TIBC), and unsaturated iron-binding capacity (UIBC), between control and K7174-treated mice (Table S3). It is possible that our observation period was short, and thus a relatively small decrease in hepcidin level (Figure 6B) may not be sufficient to demonstrate statistical significance. Alternatively, K7174 may be more efficient when administered under inflammatory conditions rather than normal conditions, as K7174 was originally developed as an antiinflammatory drug [11]. In support of this suggestion, Imagawa et al. [12] demonstrated that intraperitoneal injection of K7174 significantly ameliorated anemia triggered by coadministration of IL-1beta and TNF-alpha, whereas serum hemoglobin level was not significantly increased by administration of K7174 alone. Further analyses are required to address these questions using an *in vivo* model of ACD.

In summary, our *in vitro* and *in vivo* analyses suggested that K7174 may suppress hepcidin expression, at least in part, through modulating GDF15 expression. ACD is associated with significant morbidity and poor quality of life [5], and the amelioration of anemia may improve clinical outcomes of these patients. As it is often difficult to correct the underlying disease and currently available treatments have limited success, hepcidin-lowering agents [28,29], including K7174 or perhaps orally administrable K11706 [30], may be considered a new class of drugs in future.

## Supporting Information

Figure S1 **GATA-4 occupancy in the *HAMP* promoter is unaffected by K7174 treatment in HepG2 cells.** Quantitative ChIP analysis in control (DMSO) or K7174-treated HepG2 cells to detect GATA-4 chromatin occupancy in the HAMP promoter (*n* = 3, mean ± SE).
(EPS)

Figure S2 **Deletion for C/EBP binding sites significantly decreases *GDF15* promoter activity in HepG2 cells.** Transient human *GDF15* gene promoter analysis in HepG2 cells. We deleted -110/-103 or -87/-80 C/EBP binding region of the GDF15 promoter construct, and the luciferase activity was assayed (*n* = 3, mean ± SE, * *P* < 0.05).
(EPS)

Figure S3 **BMP4 induces *HAMP* expression via activating SMAD signaling in HepG2 cells.** (A) Western blotting analysis of whole-cell extracts from BMP4-treated HepG2 cells. Human recombinant BMP4 was treated at 0, 10 and 100 ng/mL for 30 min. Anti-phospho-SMAD1/5/8 and SMAD1 antibodies were used. Alpha-Tubulin was used as a loading control. (B) Quantitative RT-PCR validation of array results for *HAMP* gene. The expression level of each target gene relative to that of *GAPDH* was calculated (*n* = 3, mean ± SE, * *P* < 0.05). The expression level of BMP4-untreated control cells was set to 1.
(EPS)

Table S1 **Oligonucleotide primers.**
(EPS)

Table S2 **Expression profiling data by K7174 treatment in HepG2 cells.** Genes showing > 1.5-fold activation or repression are shown. We set a cut-off value of > 100 based on the expression signal.
(XLSX)

Table S3 **Biochemical analysis for K7174-treated mice.**
(EPS)
